# Genomic insights into the complex demographic history and inbreeding phenomena during Zhou Dynasty on the Central Plains of China

**DOI:** 10.3389/fmicb.2024.1471740

**Published:** 2024-09-13

**Authors:** Xiyan Wu, Baoxu Ding, Linyi Nie, Canshuo Zhong, Pengxiang Liu, Jingteng Liang, Lin Wang, Xiangping Gao, Jiyin Wei, Yawei Zhou

**Affiliations:** ^1^School of History and Culture, Henan University, Kaifeng, Henan, China; ^2^The Biological Archaeology Laboratory, Henan University, Kaifeng, Henan, China; ^3^School of Life Sciences, Zhengzhou University, Zhengzhou, Henan, China; ^4^School of Archaeology and Cultural Heritage, Zhengzhou University, Zhengzhou, China; ^5^School of History and Culture, Hubei University, Wuhan, Hubei, China

**Keywords:** ancient DNA, genetic diversity, social structure, inbreeding, Zhou dynasty, Chinese civilization

## Abstract

In the Central Plains of China during the Zhou Dynasty (1046-256 BCE), the social hierarchy gradually solidified, accompanied by frequent wars and the phenomena of multicultural and multi-ethnic integration. These social phenomena collectively influenced the population’s genetic structure at that time. However, our understanding of the genetic history of this period remains largely unknown owing to limited ancient DNA studies. In this study, we successfully obtained 11 ancient genomes from the Guanzhuang site during the Zhou Dynasty on the central plain of China. Our findings revealed remarkable genetic continuity with the Neolithic populations of the Yellow River Basin and emphasized genetic diversity through the analysis of uniparental genetic markers. Population structure analysis further confirmed the genetic similarity between the Guanzhuang population and ancient populations of the Yellow River Basin and indicated genetic exchanges with ancient populations from surrounding regions. Intriguingly, signs of inbreeding within the Guanzhuang community cast doubt on the stringent enforcement of the contemporary marital regulations against consanguineous marriages within the same surname or clan. These revelations significantly enhance our insight into the complex interplay of ancient demography and societal organization, concurrently presenting a genetic perspective to view the complex evolution of Chinese civilization’s multiethnic.

## 1 Introduction

The Central Plains generally refer to the mid and lower reaches of the Yellow River Basin. This location served as a hub for agricultural domestication in ancient China and as the cradle of the ancient Chinese civilization (Zhang et al., 2023). Significant ancient cultures flourished in this area, including the Yangshao culture with its painted pottery, the Longshan culture with its black pottery, and the succeeding Xia, Shang, and Zhou civilizations (Li, 2002). The Central Plains region has not only witnessed the evolution and prosperity of ancient society but has also become an important area for multi-ethnic and multi-cultural exchanges and interactions (Han, 2015). With the rapid development of ancient DNA technology, researchers have been exploring the population’s genetic structure, migration patterns, and cultural interactions in the region by combining the information from ancient DNA and archaeological discoveries. For instance, a certain degree of genetic continuity between populations in the Central Plains during the Middle and Late Neolithic has been confirmed (Ning et al., 2020; Su et al., 2024). Meanwhile, genetic components from Southern China and Southeast Asia increased after the Middle Neolithic period (Ning et al., 2020; Gao and Cui, 2023). Mitochondrial DNA evidence and archaeological studies have also highlighted interactions between the Neolithic populations of the Central Plains and neighboring populations (Miao et al., 2021). However, the genetic differences among populations from different regions and eras in the Central Plains of China, especially the genetic structure of ancient populations during the Bronze Age, as well as their interactions with surrounding populations remain unclear.

During the Zhou Dynasty, there were major changes in social structure, intensified social class differentiation, the shift of dynastic centers, extensive population migrations, and increased interaction with surrounding populations. These changes laid a solid foundation for the development of ancient Chinese civilization (Pletcher, 2011). Besides, the Zhou Dynasty also formulated strict ritual systems, such as a marriage system that prohibited marriage between individuals with the same surname or clan, to mitigate the harmful effects of consanguineous marriage (Wang, 2012). Previous studies have found that inbreeding phenomena had already appeared in the Central Plains more than 4,000 years ago (Ning et al., 2021). However, it remains unclear whether this phenomenon decreased or disappeared due to strict restrictions on the marriage system during the Zhou Dynasty. Based on that, investigating the genetic structure of the population in the Central Plains during the Zhou Dynasty is crucial to a comprehensive understanding of population changes and social structure in the Bronze Age.

In this study, we successfully obtained 11 ancient genomes from the Guanzhuang site, which is an important archaeological site during the Zhou Dynasty in the Central Plains of China (Chen and Liu, 2014). Through comprehensive ancient DNA analyses and integration with previously published ancient genomic data from the region, we aim to shed light on the demographic history of the Zhou Dynasty and the occurrence of consanguineous marriage. Our findings underscore the genetic continuity between the Guanzhuang population and Neolithic populations in the Central Plains, while demonstrating considerable genetic diversity. Finally, our study provides genetic evidence for inbreeding in the Guanzhuang population, offering new perspectives for understanding the Zhou Dynasty social structure, marriage practices, and their potential consequences.

## 2 Materials and methods

### 2.1 Archaeological context

**Figure 1:**
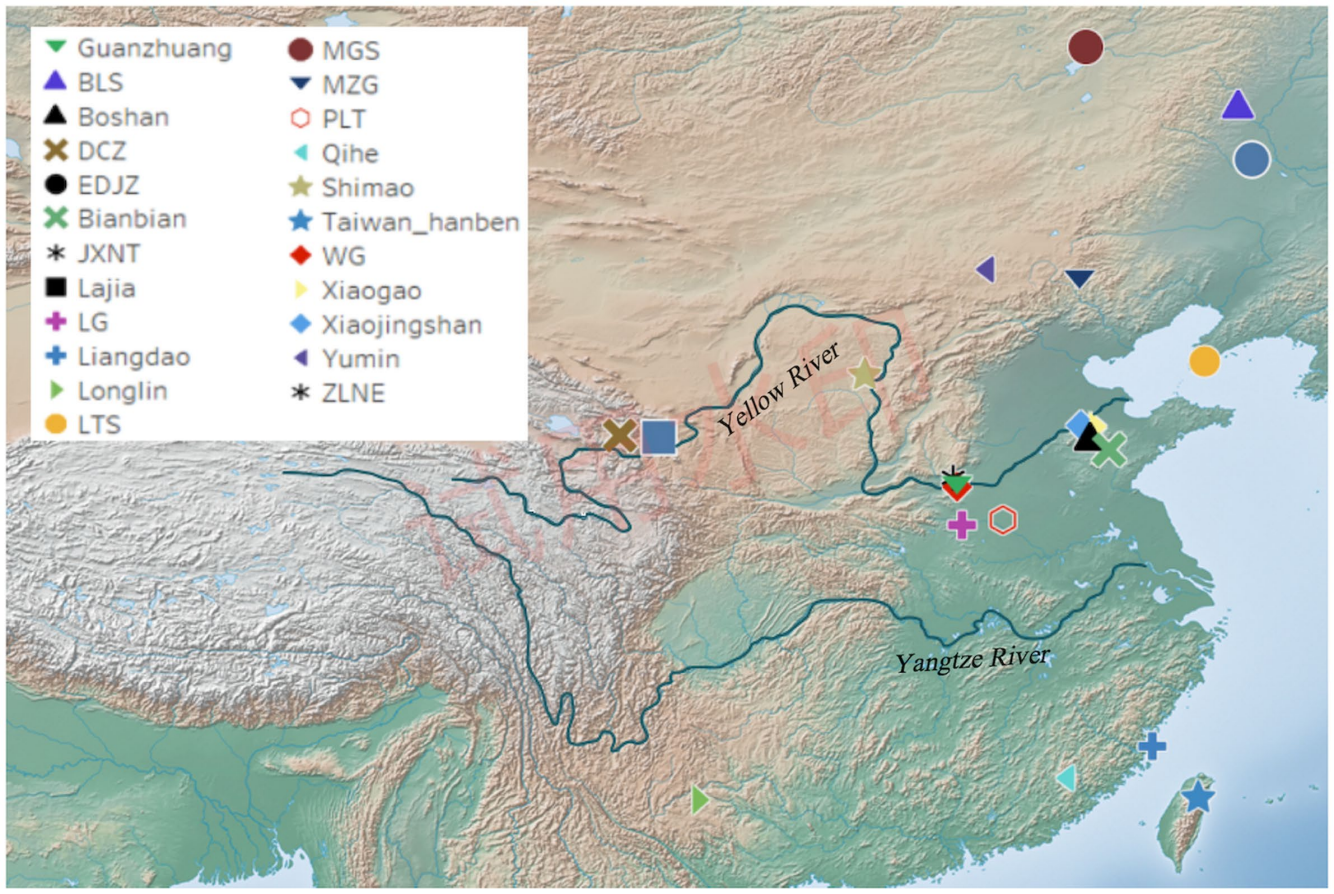
Geographic location of the Guanzhuang site and published relevant sites in China. Detailed information on previously published relevant sites is provided in Supplementary Table S1J.

The Guanzhuang site (34°51′03”N, 113°22′32″E) is located in the Guanzhuang village, Gaocun Town, Xingyang City, Henan Province, China (Figure 1). From 2010 to 2014, the History School of Zhengzhou University conducted extensive excavations, revealing the structure of this site characterized by a “convex” layout with a large and a small city (Supplementary Figure S1) (Chen and Liu, 2014). Notably, the Guanzhuang site was confirmed as the world’s oldest coin-making workshop site based on C14 dating, with coin production activities dating back to between 640 BC and 550 BC (Zhao et al., 2021). Further excavations in 2018 uncovered a wealth of relics, including tombs, pottery kilns, and ash ditches affirming the site’s significance during the Zhou Dynasty (Tao and Zhang, 2019). We collected teeth and petrous bones from 11 individuals for ancient DNA analysis.

### 2.2 Ancient DNA extraction, library preparation, and sequencing

Ancient DNA extraction and library preparation were carried out in the dedicated ancient DNA facility at Henan University in China, following the established protocols (Yang et al., 1998; Ning et al., 2020). The human remains were cleaned with 75% ethanol, then washed with 5% NaClO, and finally irradiated with UV light for 30 min on each side. The tooth sample was drilled using a dental drill (Strong 90) to obtain powder. For the petrous bones, we used a sandblaster machine (Renfert, Germany) to remove the external parts while preserving the dense area around the cochlea. Subsequently, we ground the cleaned inner part into a fine powder using an automatic grinding instrument (JXFSTPRP-24L, Shanghai Jingxin Industrial Development Co., Ltd.). 50–120 mg of bone powder was collected for ancient DNA extraction following Dabney’s protocols (Dabney et al., 2013). Double-stranded libraries were prepared following a published protocol but with minor modifications (Meyer and Kircher, 2010). Briefly, 20 μL DNA extract was prepared for the blunt-end-repair step by adding T4 polymerase and T4 PNK, followed by Qiagen MinElute purification. The adapter mix was added onto the blunt-ends using Quick Ligase followed by Qiagen MinElute purification. The adapter fill-in step was performed using Bst DNA polymerase, followed by incubating at 37°C for 30 min and inactivation at 80°C for 20 min. Libraries were amplified with dual-indexing primers (P5 and P7) using Q5 High-Fidelity DNA polymerase and then purified using Agencourt AMPure XP Bead (Beckman Coulter). We qualified the final libraries by Qubit 4.0 (Thermo Fisher) and then sequenced the libraries on an Illumina NovaSeq 6,000 platform at the Novogene Company, in China.

### 2.3 Sequence data processing and genotyping

The raw data was processed using the EAGER pipeline (Peltzer et al., 2016). We clipped the adapters and merged the paired-end reads into single-end sequences using AdapterRemoval v2.3.1 (Schubert et al., 2016), then only those 30 bp or longer collapsed reads were mapped onto the human reference genome (hs37d5) using BWA v0.7.17 (Li and Durbin, 2009) with the parameter “-n 0.01.” PCR duplicates were removed using Dedup v0.12.8, and we retained only those uniquely mapped reads that had a Phred-scaled mapping quality score of 30 or above utilizing samtools v1.17 (Danecek et al., 2021). We validated the data by analyzing the post-mortem chemical alterations in ancient DNA using mapDamage v2.2.2 (Jónsson et al., 2013). Furthermore, we estimated the mitochondrial contamination levels employing Schmutzi (Renaud et al., 2015) and assessed nuclear genome contamination rates for males specifically by analyzing the X chromosome with the ANGSD v0.940 (Korneliussen et al., 2014). We also employed the mitochondrial-to-nuclear DNA (mt/nc) ratio to evaluate contamination. When the mt/nc ratio is below 200, the contamination rate of mitochondrial DNA can serve as a reliable indicator to reflect the contamination rate of nuclear DNA in the sample (Furtwängler et al., 2018). To minimize biases resulting from the postmortem DNA damage, we masked the first and last ten bases of each read using bamUtils v1.0.15 (Jun et al., 2015) with the option “trimBam.” We used SAMtools mpileup with “-q30, −Q30” parameters and pileupCaller^1^, with “--randomHaploid” option for pseudodiploid genotype determination, utilizing a random sampling strategy from the “1,240 k” SNPs set (Mathieson et al., 2015). Subsequently, we integrated the newly generated genotype data with worldwide genotype datasets of present-day and ancient individuals, sourced from the Affymetrix “Human Origins” (HO) panel (597,573 SNPs) and the “1,240 k” panel (1,233,013 SNPs). The two datasets were obtained from the Allen Ancient DNA Resource (AADR) v50.0^2^ (Mallick et al., 2024).

### 2.4 Sex determination and uniparental haplotype assignment

The genetic sex of individuals from the Guanzhuang site was determined by comparing the coverage of the X and Y chromosomes. In males, the coverage of the Y chromosome is comparable to the coverage of the X chromosome, approximately half of the autosomes. Whereas, in females, the coverage of the Y chromosome is close to zero and the coverage of the X chromosome is roughly equal to that of the autosomes (Fu et al., 2016). To ascertain the mitochondrial DNA haplogroup, we realigned the trimmed collapsed reads mapped onto the human mitochondrial reference genome (NC_012920.1). The mitochondrial consensus sequences were generated using the log2fasta program in the Schmutzi package and then assigned mitochondrial haplogroup using HaploGrep v2.4.0 (Weissensteiner et al., 2016). We employed the scripts “Yleaf.py” and “predict_haplogroup.py” within the established Yleaf software (Ralf et al., 2018) suite to accurately delineate the Y-chromosome haplogroup, generating an output that encompasses both ancestral and derived single-nucleotide polymorphisms (SNPs) of the Y-chromosome phylogenetic tree. Additionally, we conducted a rigorous visual inspection of these critical SNPs using the Integrative Genomics Viewer (IGV) software to ensure their accuracy (Thorvaldsdottir et al., 2013).

### 2.5 Genetic relatedness and ROH analysis

Considering the extremely low coverage and a mitochondrial contamination rate exceeding 5% observed in GZM25-1, this individual was excluded from the kinship analysis. We applied three different methods including READ (Monroy Kuhn et al., 2018), KIN (Popli et al., 2023), and PMR (Kennett et al., 2017) for assessing the genetic relatedness of the remaining ten Guanzhuang individuals. The READ method computes the proportion of non-identical alleles in non-overlapping 1 Mb segments of the genome between samples, and is designated as P0. To account for the diversity within the target population, this P0 value is standardized against the expected value from a randomly selected pair of unrelated individuals from a comparable population. Lower P0 scores suggest a higher level of shared chromosomal segments. The KIN method employs a Hidden Markov Model to estimate the relatedness of individuals from identity-by-descent fragments, enabling precise identification of up to 3rd-degree relatives and discrimination between sibling and parent–child relationships even with minimal 0.05× coverage. The PMR value is obtained by comparing allele variations at each genetic locus between individuals and normalizing this count by the total number of loci with available data for both. Furthermore, to gain insights into the level of inbreeding, we employed hapROH^3^ to identify genetic segments lacking heterozygous sites, thereby estimating the extent of inbreeding through the distribution analysis of these ROH (regions of homozygosity) segments (Huang and Ringbauer, 2022).

### 2.6 Population structure analysis

We initially performed principal component analysis (PCA) on the HO dataset using smartpca v18140 implemented in EIGENSOFT package (Patterson et al., 2006) with the option “lsqproject: YES “and “shrinkmode: YES.” In total, we calculated principal components (PCs) for two sets of present-day populations: Eurasian populations and East Asian populations. Ancient samples were projected onto the first two principal components calculated by the present-day populations. PCA results were visualized by the ggplot2 package in the R software^4^. We also performed an unsupervised admixture analysis using ADMIXTURE v1.3.0 (Alexander et al., 2009) after removing SNPs with minor allele frequency lower than 1% (--maf 0.01) and pruning for strong linkage disequilibrium in Plink v1.90 (Chang et al., 2015) with the parameters “--indep-pairwise 200 25 0.2″. We ran ADMIXTURE for *K* values from two to twenty with fivefold cross-validation (--cv. = 5). We identified the best run based on the lowest cross-validation error, subsequently visualizing the inferred ancestry components by the AncestryPainter tool (Feng et al., 2018).

### 2.7 Allele sharing analysis

The outgroup f3-statistics and f4-statistics were computed using the qp3Pop v651 and qpDstat v980 programs in ADMIXTOOLS to explore the shared genetic drift between the Guanzhuang population and other ancient and modern populations (Patterson et al., 2006). The outgroup f3-statistics were performed in the form of f3 (Mbuti; X, Guanzhuang) using the parameter “inbreed: YES,” here we used the Mbuti population from central Africa as an outgroup population and X represented by the Eurasian population. The top 30 populations with more than 10,000 SNPs ranked by F3 values are visualized using the DataGraph v4.5.1. The f4-statistics were performed in the format of f4 (Mbuti, A; Guanzhuang, B) with the parameter “f4mode: YES” to explore the additional gene flow between A and B/Guanzhuang. Here, we selected ancient and modern Eurasian populations to represent population A, while ancient populations from various regions of East Asia were chosen as population B. We also evaluated f4 statistics of the form f4 (Mbuti, YR-related; Guanzhuang, ancient Eurasians) to test whether ancient populations from the Yellow River Basins share more ancestry with Guanzhuang populations. Standard errors are calculated by 5 cM block jackknifing as implemented in f3-statistics and f4-statistics.

### 2.8 Admixture modeling

We performed the admixture modeling using qpAdm v1520 from the ADMIXTOOLS package with the parameters “allsnp: YES” and “details: YES” to investigate the corresponding admixture sources and admixture proportions of the Guanzhuang population as well as one outlier of Guanzhuang population (Patterson et al., 2006). We selected source populations for our admixture modeling based on the results of PCA, F statistics, and previously published studies. Specifically, we used a set of 12 populations as the base outgroups, such as Central African hunter-gatherers (Mbuti.DG), indigenous Andamanese islanders (ONG.SG), Native Americans (Mixe.DG), Neolithic farmer from Levant (Israel_Natufian_published), Neolithic farmer from Iran (Iran_GanjDareh_N), Epipaleolithic European Hunter-Gatherer (Italy_North_Villabruna_HG), Neolithic farmers from the Anatolia (Turkey_N), Epipaleolithic Siberia Hunter-Gatherer (Russia_Ust_Ishim.DG), indigenous people of New Guinea (Papuan.DG), the oldest modern individual in Europe (Russia_Kostenki14), Southern East Asian-related ancestry (Taiwan_Hanben_IA), and early Neolithic population from Shandong (Shandong_EN). To compare the competing admixture models, we employed a rotation strategy, adding source populations to the base outgroup set one by one and repeatedly executing qpAdm (Harney et al., 2021).

## 3 Results

### 3.1 Ancient genome data from the Guanzhuang site

We extracted genomic DNA of the 11 individuals from teeth and petrous bones. Utilizing shallow shotgun sequencing, all individuals with sufficient human endogenous DNA (ranging from 3.82 to 79.76%) were further sequenced, resulting in a low autosome coverage of 0.017X–0.953X (Table 1). We checked the authenticity of ancient genome data by several methods. Firstly, all samples exhibited postmortem chemical damage patterns and possessed short average fragment length characteristics of ancient DNA (Supplementary Figure S2; Supplementary Table S1A). Secondly, we estimated mitochondrial contamination, the majority of individuals showed low-level contamination (<5%) except for GZM25-1 (6.4% contamination). Thirdly, for males, we estimated the nuclear contamination based on the X chromosome, all males had less than 5% contamination rates. Subsequently, we integrated the genomes of ten individuals (excluding GZM25-1) with two published reference datasets: the 1,240 k and HO datasets, to facilitate population genetic analysis. Ten individuals encompassed 18,857 to 417,696 SNP sites that overlapped with the 1,240 k SNP panel (Table 1).

**Table 1:** A summary of Guanzhuang samples reported in this study.

Sample ID	Skeletal element	Library length	Genetic sex	Autosomal coverage	MtDNA haplogroup	Y haplogroup	mt-contam	X-contam	1,240 k SNPs
GZH2266	Petrous	77.66	Male	0.2375	D5a2a1 + @16,172	Q1a1a	0.017	0.016023	121,965
GZM10	Tooth	62.87	Female	0.0251	Z4a1a	#	0.02	–	18,857
GZM19-2G	Petrous	57.83	Male	0.2819	M7b1a1a1	Q1a1a	0.011	0.0034	213,452
GZM25-1d	Tooth	53.75	Male	0.017	G1c1	NA	0.064	0.04496	13,187
GZM26	Tooth	55.2	Male	0.1079	Z3	O2a2b	0.038	0.02700243	76,239
GZM27	Tooth	58.09	Female	0.0972	G3a2 + 152	#	0.01	–	66,364
GZM43K	Tooth	58.27	Female	0.0214	M20*	#	0.014	–	19,672
GZM5G	Petrous	56.39	Male	0.4924	D5a2a1 + @16,172	C2b1b	0.01	0.002684	292,496
GZM76	Petrous	63.37	Male	0.9527	D5b1c	N1a	0.01	0.007655	417,696
GZW10	Petrous	52.15	Female	0.1634	B4d2	#	0.039	–	116,097
GZW29erc	Petrous	54.01	Male	0.2356	M11c	N1a2a	0.008	0.008307	163,457

### 3.2 Uniparental genetic analysis

We successfully assigned mitochondrial DNA (mtDNA) haplogroups to all individuals. A total of 10 macro mtDNA haplogroups were detected, including D (D5a2a1 + 16,172, D5b1c), *Z* (Z4a1a, Z3), G (G1c1, G3a2 + 152), B4d2, M7b1a1, M20*, and M11c, which are commonly found in East Asia. Among them, haplogroup D, G, B4, and *Z* were also identified in ancient populations from northern China (Xue et al., 2022), whereas the haplogroup M7b1a1 was found in ancient Guangxi populations in southern China (Wang et al., 2021). These findings suggest that the Guanzhuang site exhibits a high level of maternal genetic diversity. Notably, haplogroup D5a2a1, G1c1, and B4d2 were discovered in the populations of the Yangshao and Longshan cultures in the Central Plains region (Ning et al., 2020; Xue et al., 2022), indicating that the Guanzhuang population might share a maternal genetic relationship with those from the Neolithic period in this region. Six male individuals were successfully identified with Y-chromosome haplogroups, all of which belonged to the East Eurasian types (Q1a1a, O2a2b, C2b1b, and N1a), exhibiting high paternal genetic diversity. The Y-chromosome haplogroup C was primarily found in Northeast Asia and was the dominant paternal lineage among the Tungusic-speaking ethnic groups (Liu et al., 2021). Haplogroup N was prevalent in modern populations in the northern part of East Asia (Yu et al., 2023). Meanwhile, haplogroups Q and O constituted a significant proportion of the modern Han Chinese populations (Zhang et al., 2022). These findings indicate that the Guanzhuang population has multiple paternal and maternal origins.

### 3.3 Kinship estimates and parental relatedness

**Figure 2:**
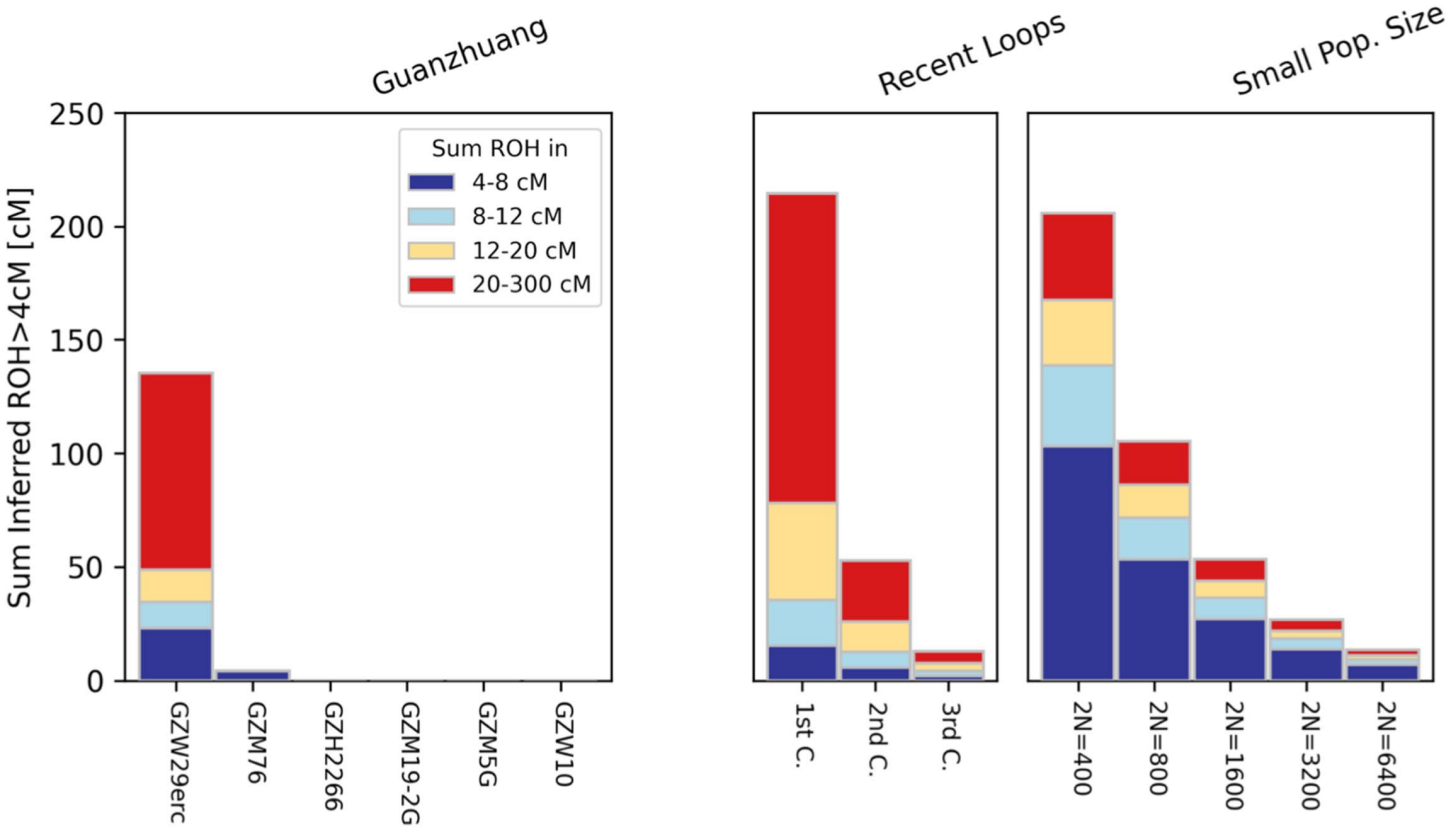
Runs of homozygosity (ROH) analysis results in Guanzhuang individuals. To ensure the robustness of the results, we restricted ROH analysis exclusively to samples containing over 100,000 SNPs overlapping with the “1,240 k” panel.

In order to ensure the accuracy of the results, we employed three different methods to estimate the genetic relatedness of ten Guanzhuang individuals, including PMR (pairwise mismatch rates), READ, and KIN. Through that, we confirmed that the ten individuals were genetically unrelated (Supplementary Figure S3; Supplementary Table S1B). Additionally, we carried out an evaluation of runs of homozygosity (ROH) to determine if there was any recent inbreeding among the Guanzhuang individuals. To ensure the robustness of the results, here we have restricted our ROH analysis exclusively to samples containing over 100,000 SNPs overlapping with the “1,240 k” panel. Our assessments revealed that two individuals (GZW29 and GZM76) had ROH segments exceeding 4 centiMorgans (cM) in length (Figure 2). More specifically, GZM76 had very little ROH overall, with the sum of all ROH segments >20 cM being zero, while GZW29 had a cumulative length of 135 cM for ROH segments (roh > 4 cM) and a total of 86 cM for long ROH segments (roh > 20 cM). The sum and length distribution of ROHs suggested that the individual GZW29 was a descendant of second cousins (Supplementary Figure S4; Supplementary Table S1C). Previous studies have revealed that the phenomenon of consanguineous marriage did occur in the Late Neolithic period in the Central Plains region, albeit infrequently (Ning et al., 2021). Nevertheless, the persistent occurrence of such marriages at this site indicates that the marital practices of “no marriage within the same surname or clan” during the Zhou Dynasty were not strictly adhered to here.

### 3.4 The genetic structure of the Guanzhuang population

**Figure 3:**
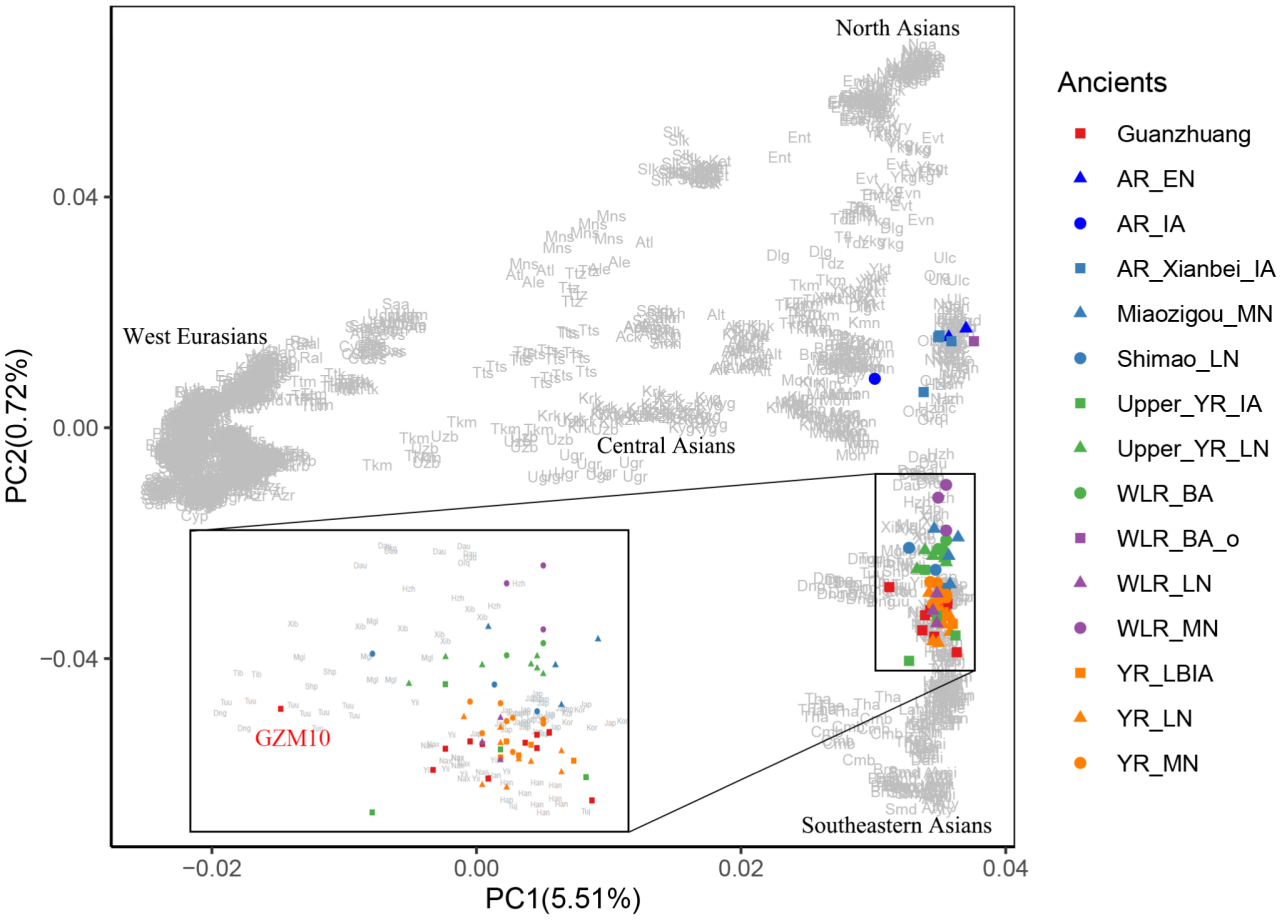
PCA analysis for Guanzhuang individuals. Ancient genomes from Guanzhuang and other relevant sites from northern China were projected onto the top PCs defined by present-day Eurasians. The population labels of present-day individuals are provided in Supplementary Table S1K.

**Figure 4:**
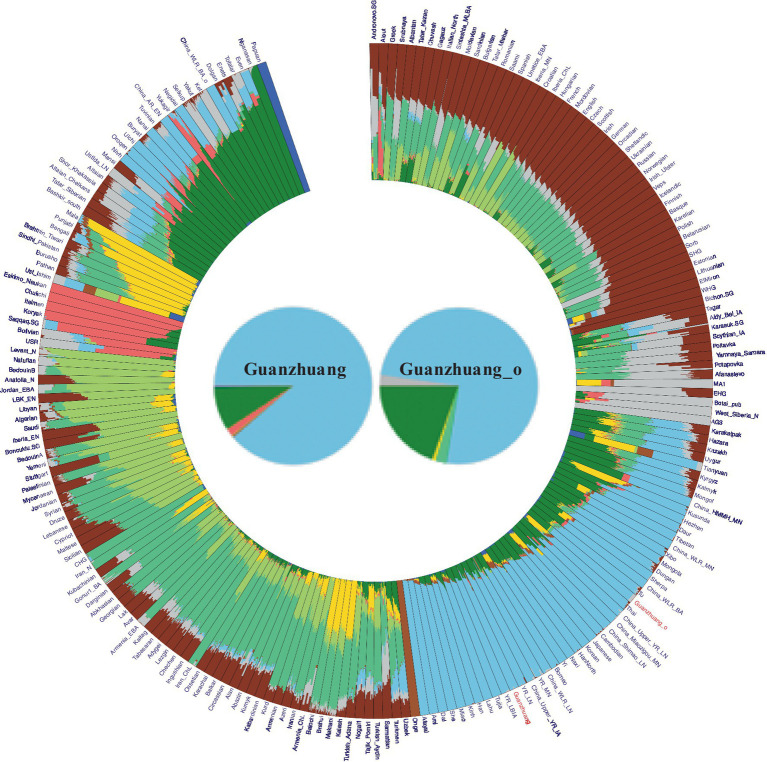
Admixture results for the “HO” dataset at *K* = 10 (as the corresponding cross-validation error was the lowest). Our results indicate that the Guanzhuang population shares a similar genetic profile with populations in the Yellow River basins, primarily exhibiting two ancestries: Northern East Asian (green) and Southern East Asian (light blue). Notably, the outlier within the Guanzhuang population exhibits a higher proportion of Northern East Asian ancestry.

To characterize the genetic profile of the Guanzhuang individuals, we initially performed principal component analysis (PCA). This involved projecting our newly sequenced and previously published ancient genomes from the Central Plains of China onto a background framework encompassing modern Eurasian populations. We found that most Guanzhuang individuals cluster closely with ancient populations in the middle to lower Yellow River Basin and modern Northern Han Chinese (Figure 3). Notably, one individual (GZM10) showed a slight deviation towards the western group on the first principal component (PC1) compared to this Yellow River-related cluster. To further refine our analysis, we conducted an additional PCA focusing on East Asian genomes. This revealed that most Guanzhuang individuals exhibit strong genetic similarities to ancient populations dating from the Bronze Age to the Iron Age of the Central Plains of China (YR_LBIA) as well as modern Northern Han populations (Supplementary Figure S5). In contrast, GZM10 deviated from this cluster, and we therefore designated this individual as Guanzhuang_o. Furthermore, our model-based unsupervised ADMIXTURE simulation analysis provided a comprehensive view of the ancient and modern Eurasian structure. The analysis revealed a pattern consistent with the results of PCA, indicating a significant genetic similarity between the Guanzhuang population and ancient individuals from the Yellow River basin (*K* = 10, with the lowest cross-validation error, Supplementary Figure S6). Additionally, the Guanzhuang_o exhibited a more genetic component related to the Northeast Asian group (Figure 4).

**Figure 5:**
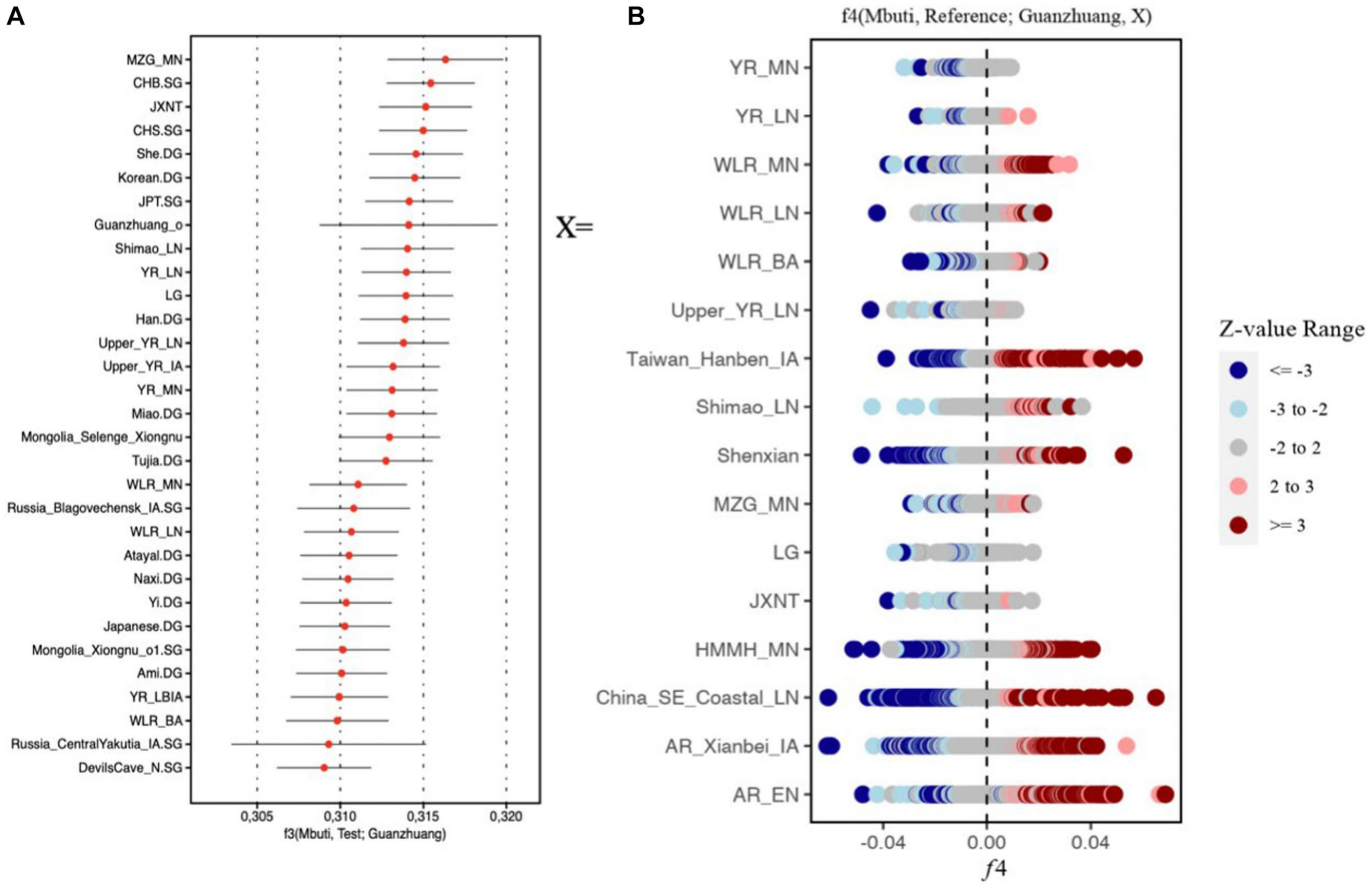
*F* statistics of Guanzhuang population. **(A)** Outgroup f3-statistics in the form f3 (Mbuti; Test, Guanzhuang) based on the 1,240 k dataset. The top 30 populations exhibiting the highest genetic drift with the Guanzhuang populations were presented. **(B)** f4-statistics test in the format of f4 (Mbuti, Reference; Guanzhuang, X) to evaluate the genetic divergence between the Guanzhuang population and various ancient populations of China. |*Z*| ≥ 3 was colored by dark red or dark blue, 3 > |*Z*| ≥ 2 was colored by light red or light blue. The detailed results can be found in Supplementary Table S2.

Similar results could also be observed in the outgroup f3 (Mbuti, Test; Guanzhuang) analysis. The larger value of the f3 statistics indicated that the Guanzhuang population shared more genetic drift with the test population after the separation from an African Mbuti outgroup. We found that the Guanzhuang population shared more alleles with the ancient population from the Yellow River Basin (YR-related populations), including Miaozigou_MN, Shimao_LN, YR_MN, YR_LN, YR_LBIA (JXNT and LG), as well as with modern Han populations (Figure 5A and Supplementary Table S1D). In contrast, the Guanzhuang outlier (Guanzhuang_o) showed the largest f3 value with China_WLR_BA_o (Supplementary Figure S7), which was modeled as deriving 93.8–100% ancestry from ANA-related (Ancient North Asian) populations and the rest from YR-related populations in a previous study (Zhu et al., 2024). We further evaluated the genetic relationship between the Guanzhuang population and ancient populations of the Yellow River basin by conducting the f4-statistic in the form of f4 (Mbuti, YR-related groups, Guanzhuang, ancient East Eurasians). The result revealed significant negative values, which further confirmed the genetic affinity between the Guanzhuang population and the YR-related ancestry (Supplementary Table S1E). In summary, these findings suggest the populations in the Central Plains region have maintained genetic continuity over a long period.

### 3.5 Genetic diversity in the later bronze/iron age individuals of the Central Plains

We utilized f4-statistics in the form of f4 (Mbuti, Reference; Guanzhuang, X), to evaluate the genetic divergence between the Guanzhuang population and population X. The “Reference” group included 287 ancient and contemporary populations, and population X comprised ancient populations from various regions of East Asia, encompassing both the northern and southern areas, as well as the Central Plains. We observed certain genetic differences between Guanzhuang and the selected populations (Figure 5B). For instance, when X was the Neolithic period populations from the Yellow River Basin, there was a certain degree of allele sharing between the Guanzhuang population and Southeast Asian populations (such as Ataya, Ami, Cambodian, etc.) (*Z* less than-3), indicating that the genetic affinity between ancient Yellow River Basin populations and present-day southern Chinese and Southeast Asians had been continuously strengthening after the Neolithic period, which is consistent with previous research findings (Ning et al., 2020) (Supplementary Table S2). Additionally, we found that the Guanzhuang population exhibits genetic affinity with Eurasian Steppe populations (such as Kazakhstan_Nomad_IA, Russia_Tagar, Kyrgyzstan_TianShan_Hun.SG, etc.), when compared to the previously published genomes of the Bronze Age to the Iron Age of the Central Plains in China (JXNT and LG, representing the Juozuoniecun and Luoheguxiang sites respectively). This was confirmed by performing the f4 (Mbuti, Reference; Guanzhuang, JXNT/LG) with a *Z* score less than −3 (Supplementary Table S1F). These findings revealed the complex population movements in the Guanzhuang population.

**Figure 6:**
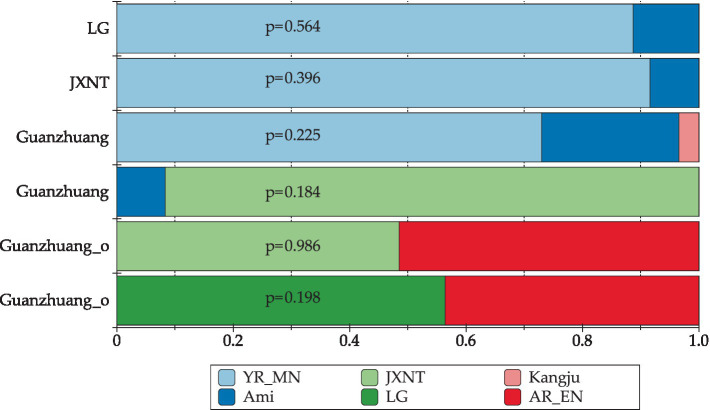
Ancestry proportions based on qpAdm analysis. The well-fitted 2-way qpAdm models for previously published YR_LBIA populations (including JXNT and LG) were presented, utilizing YR_MN and SEA-related ancestry (represented by Ami) as ancestral sources. Three-way qpAdm models quantified the genetic contribution of steppe ancestry (represented by Kangju) within the Guanzhuang population. Furthermore, an ANA-related component (represented by AR_EN) was detected in the outlier of Guanzhuang population. The comprehensive results are detailed in Supplementary Tables S1G–I.

Next, we investigated the ancestry proportions of the Guanzhuang population and the previously published genomes from the Bronze Age to the Iron Age in the Central Plains of China (JXNT and LG) using the qpAdm method. Our findings revealed that all these populations could be modeled with YR-related populations by one-way models (Supplementary Tables S1G–I). We further explored two-way models by incorporating SEA-related (Southeast Asian) populations as an additional source. The results showed that the JXNT, LG, and Guanzhuang populations could be simulated as a mixture of SEA-related and YR-related populations. Specifically, the Guanzhuang population could be modeled as deriving 77.8–91.7% ancestry from YR-related groups, with the remaining from SEA-related groups (Supplementary Table S1G). Additionally, we observed a genetic influence from the Eurasian Steppe populations through three-way models. The Guanzhuang population could be modeled as consisting of 73–78.1% YR-related populations (represented by YR_MN), 19.3–23.6% SEA-related populations (represented by Ami), and 2.6–3.4% Eurasian Steppe groups (represented by Kazakhstan_Kangju) (Figure 6). Overall, these best-fitting admixture models were consistent with the results of the f4 analysis, indicating that the Guanzhuang population may have diverse ancestral origins.

Finally, we tried to estimate the ancestry proportion of the outlier individual from the Guanzhuang site. Our findings revealed that the Guanzhuang outlier could be modeled with YR-related populations using one-way models. Notably, we also observed the influence of the ANA-related (Ancient North Asian, also known as Amur ancestry) populations on the Guanzhuang outlier, with the contribution rate of the ANA-related component ranging from 43.6–51.7%, when considering the YR-related populations as the second source (Supplementary Table S1H). Combining the results of PCA and admixture, we concluded that the Guanzhuang outlier exhibited genetic contributions from ANA-related populations compared to the other populations of the Central Plains in China. It is noted that the low genome coverage of the Guanzhuang outlier could introduce the potential for bias or spurious associations in our findings. To mitigate this limitation and reinforce our results, future studies with higher-quality samples are needed. In summary, our data suggested that the Guanzhuang population exhibited significant genetic diversity and frequent interactions with the surrounding populations during the Zhou Dynasty in the Central Plains.

## 4 Discussion

The Zhou Dynasty, a pivotal period in Chinese history spanning from 1,046 to 256 BCE, was divided into the Western Zhou and the Eastern Zhou, the latter distinguished by the Spring and Autumn Period and the Warring States Period (Feng, 2006). The Zhou’s enfeoffment system, integrating family ties with political strategy, established the Zhou king’s supremacy over vassal states (Tianyu, 2014). This era witnessed significant political restructuring and large-scale population migrations, profoundly impacting ancient China’s history and culture. The Guanzhuang site, as a significant archaeological relic, dates from the late Western Zhou period to the mid-Warring States period. The archaeological discoveries have shown that it was originally part of the Eastern Guo state in the late Western Zhou and later became part of the State of Zheng during the Spring and Autumn period (TANG et al., 2022). This offers a significant research opportunity to elucidate the dynamics of social instability and population mobility within the region through ancient genomic research.

Our analysis indicated a significant genetic continuity between the Guanzhuang population and the Neolithic populations of the Yellow River basin. Furthermore, an integrated result of mitochondrial DNA, Y-chromosome DNA, and the autosomal genome revealed the high genetic diversity within the Guanzhuang population, which is enhanced by genetic components from the southern regions of East Asia. This further supports the notion that the mixing of populations from the northern and southern parts of East Asia was intensified after the Neolithic period (Yang et al., 2020; Ning et al., 2020). These results also align with the findings of archaeological research, which demonstrate evident cultural exchange and integration between the Yellow River basin in northern China and the Yangtze River basin in the south (Dai, 2020). Since the Neolithic era, the archaeological culture of the Central Plains has progressively expanded into the Yangtze River basin, while ancient populations from the Yangtze River basin migrated northward, introducing advanced technologies such as rice cultivation and jade crafting (Song et al., 2018; Han, 2015). This bidirectional cultural interaction and population movement have significantly enhanced the cultural connotations of the Central Plains region.

In addition, we found that the Guanzhuang population might have a slight genetic affinity with the Eurasian Steppe population, harboring 2.6–3.4% of ancestral components linked to steppe-related components, as indicated by qpAdm analysis. This was further confirmed by significant negative f4 (i.e., *Z*-scores < −2.5) in the f4 statistic (Mbuti, Reference; Guanzhuang, JXNT), indicating that the Guanzhuang population exhibited gene flow with Eurasian Steppe populations, in contrast to the NXJT population (from the Juezuoniecun site dated to the Shang Dynasty). These findings could be attributed to the Zhou people’s long-term residence in an area that spans the Central Plains agricultural civilization and the northern steppe culture (Li et al., 2020; Zhou et al., 2019). The development of Zhou culture was significantly influenced by the advanced bronze civilization of the Shang Dynasty and was also characterized by continuous interactions with steppe populations, such as the Rong and Di tribes (Hui, 2013). The phenomenon of cultural integration, coupled with the evidence of genetic diversity we have discovered, substantiates the complex demographic history of the Guanzhuang site.

During the Zhou Dynasty, the social hierarchy, the system of rites and laws, and the marital system were rigorously defined (Li et al., 2020). Ancient classic documents such as “Zuo Zhuan” and “Li Ji” underscore the prohibition of consanguineous marriages within the same surname or clan to reduce the related risks caused by inbreeding (Wang, 2012; Li, 1988). However, genomic analysis of the GZW29 individual revealed the presence of long ROH, similar to those observed in second-cousin descendants (Ning et al., 2021), indicating the existence of consanguineous mating practices. Furthermore, our analysis of ROH in previously published samples from the Lusixi site in Henan Province during the Spring and Autumn Period also detected evidence of inbreeding in one individual (Supplementary Table S1C) (Maa et al., 2024). Therefore, we hypothesize that the marriage system of the Zhou Dynasty may not have been strictly enforced in this area. Additionally, anthropological studies determined that the GZW29 was a juvenile (Zhang, 2022), prompting us to speculate that the cause of death may be related to the potential hazards of consanguineous mating. To validate this hypothesis, future endeavors must involve disease association studies, particularly by using in-depth sequencing data to comprehensively elucidate the complex impacts of consanguineous marriages on human health.

In conclusion, our study provides a unique glimpse into the demographic and genetic dynamics of the Zhou Dynasty populations in the Central Plains. The observed genetic continuity, diversity, and admixture patterns, along with the evidence of inbreeding, offer valuable insights into the complex social and cultural processes that shaped ancient China. These findings not only enhance our understanding of ancient population structures but also provide a deeper context for the historical and cultural developments in early Chinese civilization. Future research, leveraging higher coverage genomic data and broader sampling, will further elucidate the intricate population dynamics and historical processes that shaped the genetic heritage of this region.

## Data Availability

The raw DNA data reported in this paper have been deposited in the Sequence Read Archive (SRA) of the NCBI, associated with the BioProject PRJNA1131741 (https://www.ncbi.nlm.nih.gov/bioproject/PRJNA1131741/).

## Footnotes

^1^ https://github.com/stschiff/sequenceTools

^2^ https://reich.hms.harvard.edu/allen-ancient-dna-resource-aadr-downloadable-genotypes-present-day-and-ancient-dna-data

^3^ https://pypi.org/project/hapROH

^4^ http://www.r-project.org/
